# Dimensionality Reduction and Channel Selection of Motor Imagery Electroencephalographic Data

**DOI:** 10.1155/2009/537504

**Published:** 2009-06-08

**Authors:** Muhammad Naeem, Clemens Brunner, Gert Pfurtscheller

**Affiliations:** BCI Lab, Institute for Knowledge Discovery, Graz University of Technology, 8010 Graz, Austria

## Abstract

The performance of spatial filters based on independent components analysis (ICA) was evaluated by employing principal component analysis (PCA) preprocessing for dimensional reduction. The PCA preprocessing was not found to be a suitable method that could retain motor imagery information in a smaller set of components. In contrast, 6 ICA components selected on the basis of visual inspection performed comparably (61.9%) to the full range of 22 components (63.9%). An automated selection of ICA components based on a variance criterion was also carried out. Only 8 components chosen this way performed better (63.1%) than visually selected components. A similar analysis on the reduced set of electrodes over mid-central and centro-parietal regions of the brain revealed that common spatial patterns (CSPs) and Infomax were able to detect motor imagery activity with a satisfactory accuracy.

## 1. Introduction

 The aim of brain-computer interfaces (BCIs) [1–5] is to create control signals by utilizing brain patterns generated by thoughts without the aid of peripheral nerves and muscles. There is a variety of invasive and noninvasive methods available to record signals from the brain. Among the noninvasive methods, the electroencephalogram (EEG) [6] is probably the most practical method available for BCI systems due to its fine temporal resolution and inexpensive recording equipment. However, due to volume conduction through the scalp, skull, and other layers of the brain, the spatial resolution of EEG signals needs to be improved. Moreover, EEG signals are usually recorded in a high-dimensional space, and it is a well-known fact that classification rules are difficult to learn and time consuming in a high-dimensional space.

To address these issues, several techniques of spatial filtering are used such as Laplacian derivations, common spatial patterns (CSPs) [7, 8], and various independent component analysis (ICA) algorithms, for example Infomax, FastICA, and SOBI [9–11]. An important attribute of a spatial filtering method is to reduce the number of dimensions and at the same time to retain all the information necessary for classification. In this regard, CSP has an internal mechanism of reducing the dimensionality of the data and was successfully used for the classification of EEG-based motor imagery data. On the other hand, ICA has an inherent indeterminacy to order and scaling, which means that the importance of the components cannot be determined on this basis. However, spatial filtering algorithms based on ICA can reduce the dimensionality of the data by visual selection of the components [12] on the basis of time-frequency maps [13] and scalp maps [14] or by applying principal component analysis (PCA) [15] as a preprocessing step before ICA.

The response of a command-related activity, depending upon the neurophysiological signal used in a BCI application, can be mapped to distinct areas of the brain. For example, ERD/ERS (event-related desynchronization/event-related synchronization) is dominant over the motor cortex, and visually evoked potentials (VEPs) are dominant over the occipital lobe. Since the approximate location of the activity associated with the control attempt is known a priori, an optimization of recording sites can also be done in advance. A reduced number of channels results in a low-dimensional feature vector which is advantageous in terms of better generalization and also reduces processing requirements. Clearly, channel selection is a dimensionality reduction technique motivated by physiological considerations and can be considered, like component selection methods, similar to feature selection. The advantage of utilizing these techniques lies in the fact that they propose to go a step further and also remove redundancy and noise in the reduced set of dimensions.

The goal of this study is to find the optimum number of dimensions of 22-channel EEG data which represents all the information without redundancy for the classification of four class motor imagery tasks. For this purpose, first, the dimensions were reduced by employing PCA preprocessing before ICA and then the classification accuracies were calculated. Similarly, classification accuracies were also calculated on 6 visually selected ICA components. Further, an automated selection of ICA components (Infomax) based on a variance criterion was also carried out. In addition, a subset of electrodes was manually selected, and the classification results were obtained by employing ICA as well as CSP and these results were compared with Laplacian derivations [16]. The data analysis was performed without removing any artifacts.

## 2. Methods

### 2.1. Experimental Data

**Figure 1:**
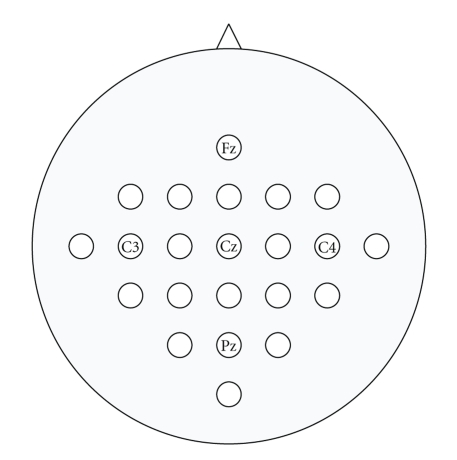
EEG electrode setup, some labels corresponding to positions in the international 10-20 system are marked. Electrodes are numbered from 1 to 22 in ascending order from top to bottom and left to right. The three subsets of electrodes used for channel selection are (1) 2–21, (2) 2–18, and (3) 2, 4, 6–14, 16, 18.

The EEG data of 4-class motor imagery was recorded with 22 electrodes placed according to the scheme in Figure 1. Monopolar derivations were used throughout all recordings (the left mastoid served as reference and the right mastoid as ground). The signals were sampled with 250 Hz and prefiltered in the range of 0.5 and 100 Hz. Furthermore, a 50 Hz notch filter was enabled to suppress line noise. The data sets were recorded from eight healthy subjects, all inexperienced in BCI training. They were sitting in a comfortable armchair in front of a computer monitor. Two sessions of each subject were recorded on different days. There were 288 trials (72 per motor imagery class) in each session distributed in a randomized order.

**Figure 2:**
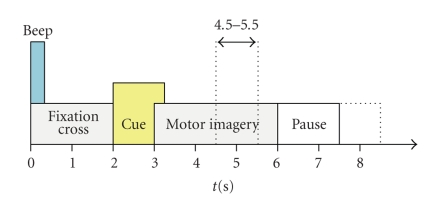
Timing of a trial of the training paradigm. The time slice between seconds 4.5 and 5.5 was used to train the classifiers. In the case of CSP the same time slice was also employed to calculate the spatial filters. However, for calculating ICA spatial filters the entire time period (0–7.5 seconds) was used.

The experimental paradigm consisted of the imagination of left hand, right hand, foot, and tongue movement. A short beep (at *t* = 0 s) along with the display of a fixation cross in the middle of the screen indicated the beginning of the trial. A visual cue (at *t* = 2 s) in the form of an arrow pointing either left, right, up, or down appeared for 1.25 seconds on the screen. Each position of the arrow required the subject to perform the corresponding imaginary movement task. The disappearance of the fixation cross (at *t* = 6 s) indicated the subject to relax. Finally, 1.5–2.5 seconds of resting period with a blank screen followed before the next trial started. The experimental paradigm is illustrated in Figure 2. 

### 2.2. Dimensionality Reduction and Channel Selection

#### 2.2.1. PCA-Based ICA Components

PCA works on the premise of uncorrelatedness and sorts the components in decreasing order of variances. That is, it accumulates as much activity as possible in the first (and then in the second, third, etc.) component, constrained by the quite unreasonable assumption that the scalp maps are orthogonal. One can immediately see two consequences of this procedure: first, the constraint of orthogonality ensures the minimization of redundancy among the components. Second, a large variance is often translated as important in the field of signal processing. Therefore, the first few components are expected to contain most of the interesting dynamics, and removing the remaining components simply implies enhancing the signal-to-noise ratio. This is in contrast to ICA, which is blind to order and scaling and hence the importance of components cannot be determined automatically.

To overcome this shortcoming, PCA is often used as a preprocessing step before ICA decomposition [17, 18]. This procedure seeks to extract independent components (ICs) from the first few principal components (PCs), implicitly assuming that the relevant brain dynamics are contained in those few components. This assumption, however, has never been verified quantitatively, at least in the cases of neuroimaging data sets. By discarding many small principal components, the risk of removing potentially interesting information cannot be neglected.

The low-variance components may not necessarily be unimportant ones, therefore choosing a certain number of PCA components for subsequent ICA decomposition is always an open question. To address this issue, the dimensions of the data sets were reduced in steps of one by applying PCA preprocessing. More specifically, each 22-channel EEG data set was first decomposed into 21, 20,…, and 6 PCA components data sets (yielding 16 data sets), respectively, before ICA decomposition. The ICA algorithms chosen for this study were Infomax, FastICA, and SOBI [9–11]. Infomax seeks to find maximally independent components, whereas FastICA maximizes the nongaussianity between the output components. SOBI (second-order blind identification), on the other hand, achieves source resolution by a simultaneous diagonalization of covariance matrices. In the case of FastICA, a tangent hyperbolic was used instead of the default nonlinearity of a third-order polynomial. Moreover, the deflationary approach was employed instead of a symmetric decomposition. In contrast, all the default parameters were used in Infomax. Similarly, SOBI was implemented by utilizing a default value of 50 time delays [12].

#### 2.2.2. Visual Selection of ICA Components

**Figure 3:**
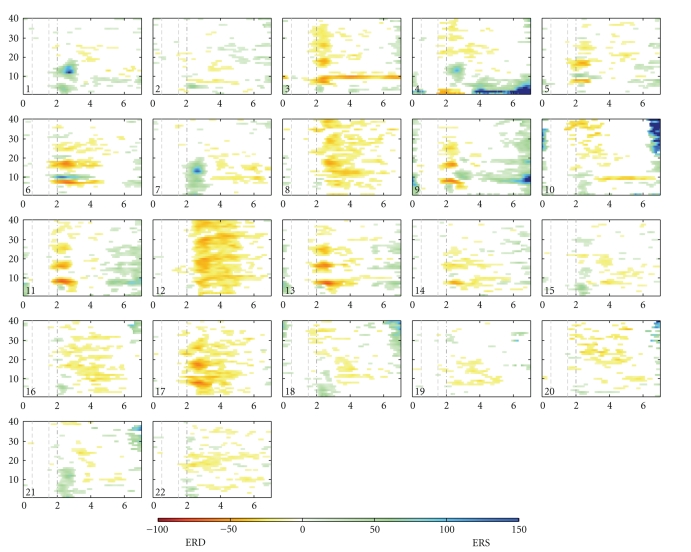
Time-frequency maps (Infomax-subject s5 session 1): components are numbered in order of mean projected variance. The six visually selected components are 5, 8, 12, 14, 16, and 18.

**Figure 4:**
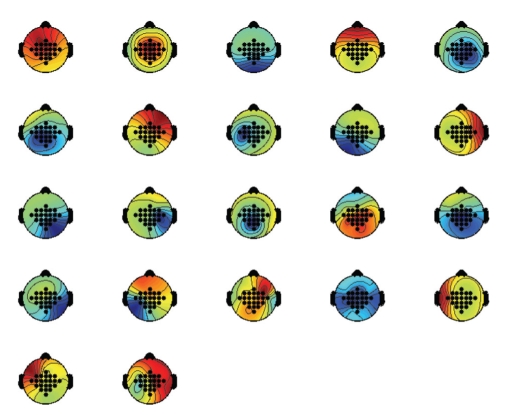
Topographic maps (Infomax-subject s5 session 1): components are numbered in order of mean projected variance. The six components (from left to right, top to bottom) selected are 5, 8, 12, 14, 16, and 18.

 The discrimination between important (task-related) and unnecessary (noisy) components in this analysis was ascertained by inspecting time-frequency maps [13] and topographic maps [14] (see Figures 3 and 4). For example, important hand imagery components were the ones focused on contralateral regions over the motor cortex area containing mu or beta ERD. The ipsilateral components containing ERS activity were also important. In the case of foot or tongue imagery, midcentral or parietal components containing localized activity were considered. The components chosen to depict tongue imagery contained dominant ERS activity, whereas for foot imagery both ERD and ERS patterns were more significant [19]. In contrast, the components that showed scattered activity over the whole surface on a topographic map (which is merely a projection of the components [**w**_1_,…, **w**_*n*_] on a two-dimensional head surface) were not chosen [12].

Summarizing, a priori knowledge of the physiological processes underlying motor imagery helped in selecting the most important components. The idea was to choose a minimum number of components to depict the suitability of ICA itself for dimensional reduction. In the selection process, care was taken to include at least one component representing each task. However, in many subjects more than one significant component corresponding to a specific task were present. This was mostly true for contralateral components and rarely for central components. Therefore, two additional components were selected, somewhat heuristically but also based on analysis [13, 14]. It should be mentioned that this selection of six components may not be so adequate for all the subjects, as each can have an individual “best-number-of-components” that represent the sources of interest. However, a fixed number of components was needed to assess the relative performances of the three ICA algorithms. Finally, the preliminary results on the average (across the subjects) were comparable with those of the full range of 22 components.

#### 2.2.3. An Automated Selection of ICA Components

 As mentioned in the introduction ICA is ambiguous to scaling as well as order. Therefore, unlike PCA importance of components cannot be determined on the basis of variance of the components. However, a mechanism of pseudo-order is available in Infomax: the components are arranged according to their mean projected variance. This implies that the first component contributes most to the power of EEG signal, the second contributes the second most, and so forth. As a consequence of this order frontal and temporal components usually find their way in the top order. Additionally, nonspecific and nonlocalized components sometimes can also be found in the upper order. This makes automatic selection of motor imagination components difficult if not impossible.

A way to tackle this issue is to employ prior information in automatic selection of components. It is a well known fact that oscillatory patterns (ERD/ERS) are pronounced in 8–14 Hz (alpha rhythm) and 14–30 Hz (beta rhythm). Due to this reason, features of data sets belonging to motor imagination experiments are usually (as is the case in this paper) extracted in 8–30 Hz. Utilizing this prior information, components were first filtered in the range of 8–30 Hz and then their variances estimated. Sorting the components in the descending order would therefore depict the potential importance of the components. This way, first six, eight, and then ten components were selected. This analysis is performed only with Infomax.

#### 2.2.4. Channel Selection

Similarly, prior information about task-related activity was used in the manual selection of a subset of the total number of 22 electrodes. For this analysis, 3 subsets of 22 electrodes were chosen (see Figure 1). The first subset consisted of electrode numbers 2 to 21, that is, the entire scalp except frontal and occipital regions. The second subset included electrode numbers 2 to 18, comprising mid-central and centro-parietal regions. Similarly, the third subset contained electrodes C3, C4, Cz, and electrodes surrounding them (in total 13 electrodes). With each of these three subsets of electrodes, spatial filters were calculated by employing three ICA methods as well as CSP. 

### 2.3. Feature Extraction and Classification

The next paragraph describes the cross-validation, feature extraction, and classification procedures for the ICA components with or without dimensionality reduction and channel selection. In each instance of a reduced number of ICA components or a subset of channels selected, the number of band power features were also different. Other than that, identical procedures were employed for 6 visually selected ICA components, automatic selection of ICA components (6, 8, and 10) and also 6 Laplacian components. The same holds for the ICA components based on manual selection of the subset of electrodes and also for components extracted after dimensionality reduction with PCA.

First, the ICA unmixing matrix was multiplied to the raw signals to extract independent components. In the next step, logarithmic band power features in the range of 8–30 Hz were calculated. For example, 22 features were computed when utilizing all the 22 ICA components. Next, a 10 × 10-fold cross-validation procedure [20] was performed. In other words, 100 different combinations of trials were created. For each of this combination, 90% of the trials were used for training four linear statistical classifiers (Fisher's linear discriminant analysis, LDA) combined in a one-versus-the-rest classification scheme [20]. Within each trial, samples between seconds 4.5–5.5 were used to train the classifiers. These classifiers were then applied to the remaining 10% of the data, and the classification accuracy was calculated by choosing the class corresponding to the maximum value of the four LDAs. The whole process mentioned above was repeated for all the 100 combinations, and the classification accuracy was calculated. The overall performance of the system was evaluated by taking an average of the classification accuracy of each combination.

Similarly, classification results of the CSP-preprocessed data (manual selection of subsets of electrodes as well as the complete set) are also presented. First, signals were bandpass filtered in the range of 8–30 Hz and then a 10 × 10-fold cross-validation procedure [20] was performed by creating 100 different combinations of trials. Each of this combination was divided into 90% and 10% portions. The (four) spatial filters were calculated on the basis of the 90% portion and were then multiplied to this data. In the next step, 6 components (the first and last three) were chosen and log-transformed normalized variances were calculated for each of the components. Next, these features were forwarded to four linear statistical classifiers (again using a one-versus-the-rest scheme). It should be noted that each classifier received the same 24 features. The classifier weights were calculated and these classifiers plus the four spatial filters were then applied to the remaining 10% of the data and the classification accuracy was calculated by choosing the class corresponding to the maximum value of the four LDAs. The whole process mentioned above was repeated for all the 100 combinations and the classification accuracy was calculated. Finally, the mean of these accuracy values was estimated. The same time slice (between seconds 4.5–5.5) was used to train the classifiers as in the case of ICA. In the case of CSP, this interval was also used to calculate the spatial filters. 

## 3. Results

**Figure 5:**
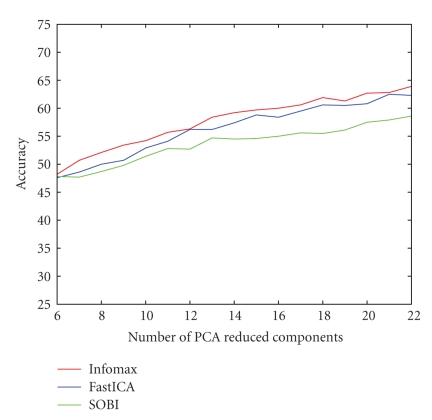
Comparison of three ICA algorithms with reduced dimensionality by PCA preprocessing in the step of one, that is, from a total of 22 components to 6 PCA reduced ICA components.

The mean accuracy values showed an almost linear increase with an increasing number of PCA-preprocessed components. This is true, in general, for all the ICA algorithms, as depicted in Figure 5. The maximum values obtained for Infomax and SOBI were 63.9% and 58.6%, respectively. In both cases, spatial filters were calculated without PCA decomposition. However, in the case of FastICA, the maximum value of 62.5% was achieved with 21 PCA-preprocessed components, which was only marginally better than the one obtained without PCA decomposition. Moreover, Infomax showed better results for each and every choice of PCA-preprocessed components in comparison to the corresponding choice with respect to FastICA and SOBI. The same is true for FastICA in comparison to SOBI. In fact, the performance of SOBI was generally poor and did not improve as markedly as Infomax and FastICA with an increasing number of PCA-preprocessed components (see Figure 5). 

**Figure 6:**
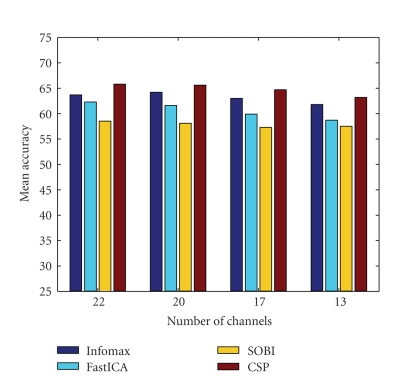
Comparison of three ICA algorithms (reduced number of electrodes) with CSP.

 One immediate conclusion that can be drawn in the analysis with manual selection of electrodes is that the spatial filters built from 20 channels (2–21) performed more or less similarly with the ones built from 22 channels (see Figure 6). More specifically, Infomax performed marginally better (64.2%) with 20 channels reduced data sets, whereas the other methods (FastICA, SOBI, and CSP) performed marginally worse with the same number of channels. Similarly, results with 17 channel data sets showed only a slight decrease in performance in comparison with either 22 channel or 20 channel data sets. In fact, the only exception was FastICA, where the performance was noticeably down by about (2.5%). However, in general, the 13 channel subset showed a pronounced deterioration for all the methods. For this analysis, CSP performed better than the rest but Infomax was only slightly worse, in comparison with CSP, for each subset of total number of electrodes considered.

**Figure 7:**
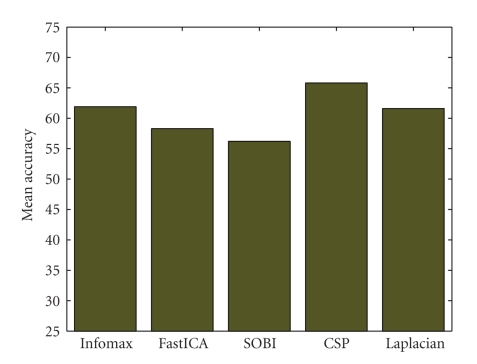
Comparison of three ICA algorithms (6 components selected by visual inspection) with Laplacian (6 components) and CSP.

For the visual selection of components with the ICA methods, Infomax (with 61.9%) performed much better than FastICA (58.3%) and SOBI (56.2%) (see Figure 7). However, CSP performed better than all methods and surprisingly, Laplacian derivations (with 6 components) performed almost as good as Infomax. In addition, the performance of Infomax with visual selection of components was better than the corresponding performance with 19 PCA-preprocessed components and only slightly worse than 21 components. Similarly, the performance of Infomax with the reduced set of 17 electrodes was only slightly better than the visually selected components by the same method. On the other hand, the result with visual selection of components of FastICA (58.3%) was comparable with 16 PCA-preprocessed components and also with the reduced set of 13 electrodes. In the case of SOBI, 19 PCA-preprocessed components performed comparably with 6 visually selected components by the same algorithm. However, the performance was comparatively lower than the reduced set of 13 electrodes. 

 The components in Figures 3 and 4 are arranged in the following order by the automatic selection procedure: 8, 12, 11, 17, 3, 13, 5, 10, 6, 16, 9, 22, 20, 18, 21, 1, 14, 7, 2, 19, 15, 4. That is, two contra-lateral components can be found among the first in this list. The frontal component (4) can be seen in the last position, whereas the temporal component (10) is at place 8. In fact, one of the temporal components was usually found to be present in the first 8 to 10 components. In few instances, components with scattered topography were also found among the first few. However, these components usually represented task-related activity. This is amply demonstrated in Table 1. The mean accuracy for 6 automatically selected components is comparable with the one for 6 visually selected components. The performance of 8 automatically selected components is even better and that of 10 components is almost equivalent to the full range of 22 components.

## 4. Conclusions

As the spatial filters built with ICA methods and CSP on the reduced set of 20 electrodes performed more or less comparably with the ones built with the total number of 22 electrodes, it can be concluded that frontal and occipital regions of the brain do not capture significant motor imagery patterns. Therefore, data recorded from these areas of the brain can safely be excluded before building spatial filters. Similarly, results with the reduced set of 17 electrodes showed that especially CSP and also Infomax were able to focus motor imagery activity in mid-central and centro-parietal regions. The exception of FastICA in this regard could be due to the algorithmic choice of the deflationary approach instead of a symmetric decomposition employed for source resolution.

The results presented for PCA-preprocessed ICA algorithms lead to the conclusion that at least 20 PCA components out of the 22 electrodes are necessary for preserving relevant information. For the 22 channels data sets considered in this analysis, PCA preprocessing was not found to be a suitable preprocessing method that could retain motor imagery information in a smaller set of components. It should also be mentioned that dimensional reduction even up to 6 PCA components still preserves about 98%–99% of the total variance in general. This fact simply implies that reducing the dimension by throwing away low-variance components more often than not results in a loss of important and relevant information.

The selection of ICA components on the basis of visual inspection of time-frequency maps and scalp maps has a subjective bias. Even this heuristic measure presented in this work outperforms the PCA-based method of dimensional reduction. Infomax in particular performed much better than the other ICA variants considered. The overall result of Infomax with 6 visually selected components was only 2.0% less than the corresponding result with all the components and about 14% higher than 6 PCA-preprocessed Infomax components. This implies that Infomax was able to incorporate most of the task-related activity in few components.

However, visual selection of components is a manual and subjective procedure. In order to automatically select these few important components, a simple variance-based criterion was employed. This procedure proved to be a success as only 8 to 10 components were needed to capture relevant motor imagination activity. Based on the relative performances, especially with reference to the PCA-based method, this procedure can be recommended as a dimensional reduction technique for motor imagery data sets.

## 5. Discussion

The variance-based procedure is applicable to motor imagery data sets. Moreover, the number of required components can further be reduced if some of the unwanted components can be located a priori. Further, a very small set of correctly selected clean components has the potential of enhancing the performance of ICA algorithms. Therefore, an improved general purpose automated method needs to be developed for the determination of important components. This objective can be achieved for example by employing some of the new ideas such as constrained ICA. Various ways to incorporate prior information in ICA algorithms have already been suggested [21, 22]. For example, reference signals of different classes can be employed as constraints in ICA algorithms. Similarly, probability distribution functions of reference signals can be incorporated directly as models in ICA algorithms. Independent components can thus be extracted under the constraint of being similar to the reference signals or their probability distribution functions. Moreover, this method is oblivious to the square mixing assumption of standard ICA [21]. Therefore, in addition to enhancing the signal-to-noise ratio of neuroimaging data, constrained ICA can also deal effectively with the issue of dimensional reduction.

The standard PCA method cannot be recommended, at least for the data sets considered in this study. This leads to the interesting question whether there are other PCA-based strategies for the selection of the components to retain the most important information. A recent paper [18] proposed an alternative signal representation that is based on PCA for dimensionality reduction and ICA conducted across all subjects and conditions simultaneously. The results based on partial least squares (PLSs) analysis showed an enhancement of the task-related activity under compression. Another approach worth implementing is nonlinear PCA [23, 24]. Nonlinear PCA can be considered as a type of ICA under special conditions [23]. The nonlinearity is introduced in the objective function, but the output variables are still a linear combination of the input variables. It can therefore be seen as an ICA with an additional advantage of PCA.

A novel method in a very recent paper [25] utilized ICA for source localization. The method then found the optimal positions of two bipolar electrodes with the purpose of reducing the number of electrodes to four. The goal was to optimize the number of electrodes for practical BCI systems. The results showed promise for two-class motor imagery data. The extension to the four-class problem, such as the data analyzed in this paper, is also possible and could be carried out in future analyses.

## Figures and Tables

**Table 1:** Infomax: mean accuracy values of automatically selected components, visually selected components, and the full range of components.

Components	6 (auto)	8 (auto)	10 (auto)	6 (visual)	22 (full)
Mean accuracy	61.4	63.1	63.8	61.9	63.9
